# Contrast-Independent Biologically Inspired Motion Detection

**DOI:** 10.3390/s110303303

**Published:** 2011-03-18

**Authors:** Birthe Babies, Jens Peter Lindemann, Martin Egelhaaf, Ralf Möller

**Affiliations:** 1 Center of Excellence ‘Cognitive Interaction Technology’, Bielefeld University, D-33594 Bielefeld, Germany; E-Mails: Jens.Lindemann@uni-bielefeld.de (J.P.L.); Martin.Egelhaaf@uni-bielefeld.de (M.E.); 2 Computer Engineering Group, Faculty of Technology, Bielefeld University, D-33594 Bielefeld, Germany; E-Mail: bbabies@ti.uni-bielefeld.de; 3 Department of Neurobiology, Faculty of Biology, Bielefeld University, D-33594 Bielefeld, Germany

**Keywords:** image motion, image contrast, motion detection, natural images, bioinspiration

## Abstract

Optic flow, *i.e.*, retinal image movement resulting from ego-motion, is a crucial source of information used for obstacle avoidance and course control in flying insects. Optic flow analysis may prove promising for mobile robotics although it is currently not among the standard techniques. Insects have developed a computationally cheap analysis mechanism for image motion. Detailed computational models, the so-called elementary motion detectors (EMDs), describe motion detection in insects. However, the technical application of EMDs is complicated by the strong effect of local pattern contrast on their motion response. Here we present augmented versions of an EMD, the (s)cc-EMDs, which normalise their responses for contrast and thereby reduce the sensitivity to contrast changes. Thus, velocity changes of moving natural images are reflected more reliably in the detector response. The (s)cc-EMDs can easily be implemented in hardware and software and can be a valuable novel visual motion sensor for mobile robots.

## 1. Introduction

When a mobile robot or an animal moves, the images of the environment move on its cameras’ sensors or on its eyes’ retinae, respectively. These image movements, termed optic flow, can be a valuable source of information about both the ego-motion of the agent and the spatial structure of the environment [1]. The optic flow generated by translatory movements reflects the distance of objects in the environment because the images of objects close to the moving observer move faster on the sensor than those of more distant objects.

Although most mobile robot systems carry at least one camera, optic flow analysis currently plays only a minor role in their control systems. Because visual motion cannot be sensed directly like luminance, it has to be computed from the spatio-temporal luminance changes in a sequence of images. Computer vision approaches to image motion estimation typically involve iterative smoothing processes which make the process computationally expensive [2,3].

In contrast to most robots, many animals use optic flow for ego-motion estimation and as an important source of information about the distances in the environment [4,5]. Behaviour control depending on visual motion perception can be observed throughout the animal kingdom.

Flying insects seem to rely almost exclusively on optical flow in tasks like obstacle avoidance and visual gaze stabilisation (see e.g., [6] for review). They seem to have developed an approach to the problem of motion detection and optic flow analysis in their tiny brain that is computationally cheap. Their local elementary motion detection circuits (EMDs) compute a direction-selective signal by comparing the time-course of the signals of pairs of adjacent photoreceptors. The resulting local motion estimates are then spatially pooled by neurons covering large parts of the visual field forming a set of time-dependent features jointly encoding the optic flow [7].

On the one hand, this biological approach is also very interesting for technical applications, because it is computationally cheap compared with computer vision algorithms. On the other hand, the signals of biological EMDs encode the image velocity in a nonlinear and ambiguous way. Their responses peak at a certain velocity and decrease for velocities beyond this optimum [8]. Further properties of at least basic EMD models make this motion detection scheme only a poor velocity sensor: (1) The response amplitudes of basic versions of the EMD depend on the global spatial frequency composition of the input image [9]. (2) The global contrast of the moving image changes the response of basic EMDs in a quadratic way [10,11]. (3) The time-dependent responses of individual EMDs show pronounced fluctuations that depend on the specific details of the pattern analysed by the EMD; these pattern-dependent fluctuations can be reduced by spatial integration over many EMDs looking at neighbouring points of the image [12]. (4) Even the time course of spatially integrated EMD outputs depends not only on pattern velocity but also on acceleration and higher-order temporal derivatives [10,13].

Control systems for mobile robots often combine the biologically inspired concept of flow-specific large-field integration with computer-vision algorithms for local velocity estimation that do not show the strong contrast and pattern dependence of EMDs [14,15]. Systems using biologically inspired EMDs were also proposed and successfully tested in simulation [16] and in hardware [17] but are limited to environments with a restricted range of textural properties [18,19].

Compared to the performance of models employing basic EMD variants, the responses of motion-sensitive neurons in the brain of insects, such as flies, after which EMDs were modelled, are much less sensitive to the pattern structure and contrast. In particular, they show the quadratic dependency on image contrast only for very low contrast values. For higher contrast values, the response does not increase with increasing contrast and the neuronal responses become less sensitive to the local contrast variations of the stimulus patterns [10,20–22]. This relative contrast independence is not the consequence of signal saturation at the level of the wide-field motion sensitive neuron, because the response can still be modulated by changing the image velocity, but of processing in the peripheral visual system [23].

To reduce the dependence of EMDs on local pattern contrast and, thus, to approximate the responses of their biological equivalents, various augmentations of EMDs were proposed. These range from simple saturating static nonlinearities incorporated into the motion detection process [10,21] to a sophisticated combination of nonlinearities and temporal filters [18,24]. The augmented models mimic the temporal properties and adaptive processes in the peripheral visual system, the motion detection process itself, and the spatially integrating wide-field motion sensitive neurons [18,24,25]. With these biologically inspired models, the relative independence of the responses of wide-field motion sensitive neurons of local pattern contrast could be explained to a large extent.

Here, we present a different augmentation of the EMD, making its response independent of local pattern contrast. This new model was developed predominantly with a focus on usability in robotics. It implements dynamic normalisation of the response amplitude of the EMD with respect to the local contrast of the input image by an approximative computation of the correlation coefficient of the signals of adjacent photoreceptors. We show that this augmentation largely reduces all modulations of the response of an EMD array unrelated to velocity, making the signals potentially more useful for the control of mobile robots.

In the following section we describe basic variants of EMDs, proposed by various authors. In Section 3 we present our approach for a novel EMD augmentation with dynamic contrast normalisation. Section 4 describes the materials and the methods we used to compare the response behaviour of basic models and augmented models. In Section 5 we present the test results from simulations based on real-world images for the different models. In Section 6 we conclude with a discussion.

## 2. Basic EMD Models

Based on behavioural experiments which analysed the turning preference of walking beetles in the presence of wide-field rotational movement, Reichardt and Hassenstein developed a computational model for motion detection in insects [26]. Variants of this model account for many response properties of motion-sensitive neurons in the insect brain (for review: [6,27]).

Motion detection seems to be based on similar computational principles across species ranging from insects to mammals [28]. Models for human motion perception can be shown to be mathematically equivalent to this elementary motion detector [29].

**Figure 1.**
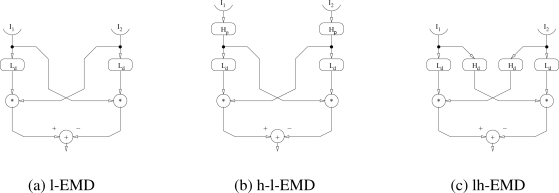
The general scheme of basic variants of elementary motion detectors. The input signals (*I*_1_, *I*_2_) originating from neighbouring points in space pass through different combinations of peripheral high-pass filters (H*_p_*) and delaying low-pass filters (L*_d_*). In the next step, the differently filtered signals of the neighbouring inputs are multiplied (*). In a final step, the signals from the mirror-symmetrical subunits of an EMD (which are most sensitive to opposite directions of motion) are combined. The simplest way is to subtract the signals from each other. **(a)** The simplest form using just the delaying low-pass filters. **(b)** An additional high-pass filter to the input channels removes the mean brightness from the input signal. **(c)** A similar behaviour can be achieved by applying a high-pass filter to the second input of the multiplication element.

In its simplest form, the EMD multiplies the signal of one photoreceptor with the delayed signal of a neighbouring one (l-EMD, Figure 1(a)). Typically, a linear temporal first-order low-pass filter is used as delay element. This simple correlation is maximal if the delay caused by the image moving from one input element to the other is perfectly matched by the delay caused by the filter in the signal pathway. Lower or higher velocities and different movement directions reduce the correlation of the signals.

To get an anti-symmetric response to motion in opposite directions and to make the detector insensitive to brightness changes that are independent of motion (so-called flicker), the response of a mirror-symmetrical circuit connected to the same input elements is subtracted (l-EMD, Figure 1(a)). The resulting EMD responds to movement in one (“preferred”) direction with a positive signal and to movement in the opposite (“anti-preferred”) direction with a negative signal. The response reaches a peak at an optimal velocity. Lower and higher velocities lead to gradually declining responses [8].

For single spatial wavelength input images (sinusoidal stripe patterns), the optimal velocity and the maximal response amplitude change with the wavelength *λ*. The peak of the averaged response occurs at an optimal temporal frequency *ω* (*i.e.*, the ratio of velocity *v* and spatial wavelength *λ*; *ω* = 2*πv*/*λ*) of the input signal ([30], see Equation (2)). Hence, the averaged response amplitude of a simple EMD does not only depend on pattern velocity, but also on its spatial properties, such as on the spatial spectrum and the contrast. The contrast dependence of a basic EMD is quadratic ([10], see Equation (2)). As a result, the basic EMD response only robustly reflects the direction of motion, while the velocity cannot be reconstructed from the signal without further augmentations [8,27].

Several augmentations extending this simplest EMD circuit have been proposed. In most cases, additional temporal filters were included in the model that lead to changes in the response properties. Two of these augmentations are shown in Figure 1(b,c). Adding a high-pass filter to the input channels of the detector (h-l-EMD, Figure 1(b)) removes the mean brightness (“DC component”) from the input signal which, if present, results in an oscillation of the response to constant velocity motion [10]. The reduction of the mean brightness can be achieved by applying a high-pass filter to the second input of the multiplication element (lh-EMD, Figure 1(c)). The response of this variant of the EMD shows an improved fit to the dynamic properties of fly motion sensitive neurons [30].

All augmentations that involve just changing the filter configuration conserve the properties of the EMD; namely that the response is ambiguous, and depends on pattern wavelength and contrast in a way that only allows the easy reconstruction of the motion *direction* from the signal.

### Mathematical Analysis of the Steady-State Response

The steady-state response of a basic EMD for stimulation with a single-wavelength sinusoidal stripe pattern can be computed analytically. Considering a sine pattern with a wavelength *λ*, an average brightness *I*, a brightness amplitude Δ*I*, and a constant velocity *v*, the time course of the input *s*(*t*) of a photoreceptor at the position *φ* can be described as:

(1) s(t)=I+ΔI⋅ sin (ωt+φ)

Where *ω* = 2*πv*/*λ* is the temporal frequency of the input signal. The linear temporal filters in the models cause a frequency-dependent damping of the amplitude *A*(*ω*) and lead to a phase shift Φ(*ω*). In the steady-state, the damping and phase shift are constant for a given temporal frequency. For the h-l-EMD (Figure 1(b)) the steady-state response 〈*R*〉 is [30]:

(2) $$\langle R\rangle =\Delta I^{2}\cdot \;\text{sin}\;\left(\frac{2\pi }{\lambda }\Delta \varphi \right)\cdot \frac{\tau_{L_{d}}\tau_{H_{p}}^{2}\omega^{3}}{\left(1+{\left(\tau_{L_{d}}\omega \right)}^{2}\right)\left(1+{\left(\tau_{H_{p}}\omega \right)}^{2}\right)}$$

Where *τ_L_d__* is the time constant of the low-pass filter, *τ_H_p__* is the time constant of the high-pass filter and Δ*φ* is the angular distance between the two detector inputs. The mean brightness *I* of the stimulus is eliminated by the peripheral temporal high-pass filter in the h-l-EMD and by the subtraction of the two mirror-symmetrical EMD subunits.

The steady-state value has a maximum at a certain *ω* = 2*πv*/*λ* irrespective of the spatial wavelength *λ* of the pattern. As a consequence, the response peaks at different velocities for stripe patterns differing in wavelength. Furthermore, the maximum amplitude changes for different choices of *λ*.

Equation (2) also reveals a square dependency on the contrast Δ*I*. Thus, without further augmentations, simple EMDs can be used merely for the detection of motion direction, but not to reliably estimate the stimulus velocity.

## 3. Correlation Coefficient Based Models

To obtain a detector with a more robust response to image velocity, the dependence on pattern contrast must be reduced. Nevertheless, the advantage of the simplicity of the EMD models should be maintained. Therefore we propose a new model variant based on the h-l-EMD and on the equation for the correlation coefficient.

For two measured signals $X_{1}=\left\{x_{1}^{t}\right\}$, *t* = 1..*T* and $X_{2}=\left\{x_{2}^{t}\right\}$, *t* = 1..*T*, where *x̄*_1_ and *x̄*_2_ are the averaged values of the signals, the empirical correlation coefficient can be calculated by

(3) $$\rho X_{1},X_{2}=\frac{\sum_{t=1}^{T}w\left(t\right)\left(x_{1}^{t}-{\bar{x}}_{1}\right)\left(x_{2}^{t}-{\bar{x}}_{2}\right)}{\sqrt{\sum_{t=1}^{T}w\left(t\right){\left(x_{1}^{t}-{\bar{x}}_{1}\right)}^{2}}\sqrt{\sum_{t=1}^{T}w\left(t\right){\left(x_{2}^{t}-{\bar{x}}_{2}\right)}^{2}}}$$

Where the weighting is usually constant, e.g., $w(t)=\frac{1}{T-1}$ (for the empirical variance and covariance). For a detector that can reflect changes in velocity in a dynamic way, the weighting function must decline for past measurements.

We assume a peripheral high-pass filter (*H_p_*) to remove the DC-component from the input signals (*i.e.*, *x̄*_1,2_ = 0). By realising the declining weighting and averaging of a continuous signal by using a low-pass filter *L_w_*, Equation (3) can be approximated by

(4) $$\rho X_{1},X_{2}=\frac{L_{w}\left(X_{1}X_{2}\right)}{\sqrt{L_{w}\left(X_{1}^{2}\right)L_{w}\left(X_{2}^{2}\right)}}$$

In an EMD, one of the input signals is delayed by a low-pass filter to compensate the delay caused by the spatial separation of the inputs. Thus we can replace *X*_1_ by *L_d_*(*X*_1_) or *X*_2_ by *L_d_*(*X*_2_). Combining the approximation of the correlation coefficient (4) and the basic EMD with mirror-symmetrical subunits leads to a new complex EMD which can mathematically be described by

(5) $$\frac{L_{w}\left(X_{1}L_{d}\left(X_{2}\right)\right)}{\sqrt{L_{w}\left(X_{1}^{2}\right)L_{w}\left({\left(L_{d}\left(X_{2}\right)\right)}^{2}\right)}}-\frac{L_{w}\left(L_{d}\left(X_{1}\right)X_{2}\right)}{\sqrt{L_{w}\left({\left(L_{d}\left(X_{1}\right)\right)}^{2}\right)L_{w}\left(X_{2}^{2}\right)}}.$$

**Figure 2.**
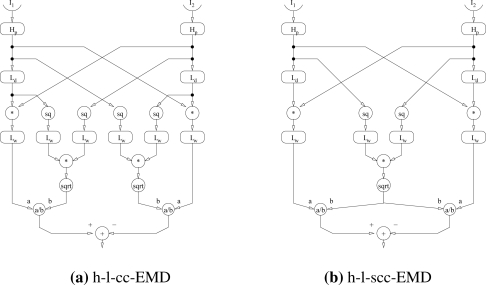
Augmented variants of elementary motion detectors. The input signals (*I*_1_, *I*_2_) originating from neighbouring points in space pass through different combinations of high-pass filter (H) and low-pass filter (L). In the next step, the differently filtered signals of the neighbouring inputs are multiplied (*) and afterwards a contrast normalisation is performed (*a/b*). The normalisation consists of a combination of square (sq), square root (sqrt) and low-pass filter. For the h-l-cc-EMD **(a)** the normalisation is based on the equation for the approximated correlation coefficient (see Equation 4). For the h-l-scc-EMD **(b)** this normalisation is simplified (see Equation 6). In a last step the signals from the mirror-symmetrical subunits of an EMD (which are most sensitive to opposite directions of motion) are combined. The simplest way is to subtract the signals from each other.

In the following we call this new model h-l-cc-EMD (correlation coefficient EMD) (Figure 2(a)). Note that the two variance terms differ only in the phase shifts caused by the low-pass filters *L_d_* of the detector. When the time constant of the low-pass filters *L_w_* approximating the integration of the variance terms is large compared to that of the delay filter, these terms are almost equal

(6) $$L_{w}\left({\left(L_{d}\left(X\right)\right)}^{2}\right)\approx L_{w}\left(X^{2}\right)$$

This observation leads us to a simplification of Equation (5)

(7) $$\frac{L_{w}\left(X_{1}L_{d}\left(X_{2}\right)\right)-L_{w}\left(L_{d}\left(X_{1}\right)X_{2}\right)}{\sqrt{L_{w}\left(X_{1}^{2}\right)L_{w}\left(X_{2}^{2}\right)}}$$

In the following we call this new model h-l-scc-EMD (simplified correlation coefficient EMD) (Figure 2(b)).

The new model maintains the ambiguity and the spatial wavelength dependency of the velocity tuning of the EMD because the correlation coefficient is still largest when the temporal delays caused by the delaying filter match those caused by the geometric separation of the inputs.

Note however, that the denominator of the fraction can be zero. With a high-pass filter in the input lines removing the DC-component of the luminance signals Equation (7), this will happen for zero velocity stimulation. Consequently, the test implementation treated this special case by returning a zero detector response if the denominator was approximately zero.

### Mathematical Analysis of the Steady-State Response

Considering the same sine pattern as used above (Equation (1)), the steady-state response of the h-l-cc-EMD is

(8) $$\langle R\rangle =\frac{T_{1}-T_{2}}{\sqrt{T_{3}\cdot T_{4}}}-\frac{T_{5}-T_{2}}{\sqrt{T_{6}\cdot T_{7}}}$$

For the h-l-scc-EMD it simplifies to

(9) $$\langle R\rangle =A_{L_{d}}(\omega )\cdot \frac{T_{1}-T_{5}}{\sqrt{T_{4}\cdot T_{7}}}$$

using the following terms for substitution:

$$T_{1}=\;\text{cos}\;\left(\Phi_{L_{d}}\left(\omega \right)+\Phi_{L_{w}}\left(\omega \right)+\frac{2\pi }{\lambda }\Delta \varphi \right)$$

$$T_{2}=\;\text{cos}\;\left(2\left(\omega t+\varphi \right)+2\Phi_{H_{p}}\left(\omega \right)+\Phi_{L_{d}}\left(\omega \right)+\Phi_{L_{w}}\left(\omega \right)-\frac{2\pi }{\lambda }\Delta \varphi \right)$$

$$T_{3}=\frac{1}{A_{L_{w}}\left(\omega \right)}-\;\text{cos}\;\left(2\left(\omega t+\varphi \right)+2\Phi_{H_{p}}\left(\omega \right)+2\Phi_{L_{d}}\left(\omega \right)+\Phi_{L_{w}}\left(\omega \right)\right)$$

$$T_{4}=\frac{1}{A_{L_{w}}\left(\omega \right)}-\;\text{cos}\;\left(2\left(\omega t+\varphi \right)+2\Phi_{H_{p}}\left(\omega \right)+\Phi_{L_{w}}\left(\omega \right)-\frac{4\pi }{\lambda }\Delta \varphi \right)$$

$$T_{5}=\;\text{cos}\;\left(\Phi_{L_{d}}\left(\omega \right)+\Phi_{L_{w}}\left(\omega \right)-\frac{2\pi }{\lambda }\Delta \varphi \right)$$

$$T_{6}=\frac{1}{A_{L_{w}}\left(\omega \right)}-\;\text{cos}\;\left(2\left(\omega t+\varphi \right)+2\Phi_{H_{p}}\left(\omega \right)+2\Phi_{L_{d}}\left(\omega \right)+\Phi_{L_{w}}\left(\omega \right)-\frac{4\pi }{\lambda }\Delta \varphi \right)$$

$$T_{7}=\frac{1}{A_{L_{w}}\left(\omega \right)}-\;\text{cos}\;\left(2\left(\omega t+\varphi \right)+2\Phi_{H_{p}}\left(\omega \right)+\Phi_{L_{w}}\left(\omega \right)\right)$$

with

$\Phi_{H_{p}(\omega )}=\text{arctan}\;\left(\frac{1}{\tau_{H_{p}\omega }}\right)$ phase response of the high-pass,
Φ_*L*_*d*__(*ω*) = *−* arctan(*τ*_*L*_*d*__*ω*) phase response of the first low-pass (delay),
Φ_*L*_*w*__(*ω*) = *−* arctan(*τ*_*L*_*w*__*ω*) phase response of the second low-pass,
$A_{L_{w}(\omega )}=\frac{1}{\sqrt{1+r_{\omega }^{2}\omega^{2}}}$ amplitude response of the second low-pass (weighting).

The equations show that the steady-state responses of the novel correlation based EMDs do not depend on pattern contrast Δ*I*. However, the term *ωt* in the variance estimates (T3, T4, T6, and T7) leads to an oscillation in the time-dependent response. This oscillation depends on *A*_*L*_*w*__(*ω*) and decreases with increasing *ω*. This oscillation has twice the temporal frequency of the input signal. The effects on the response behaviour in comparison to the basic EMD models (Section 2) are examined by simulation in the following.

## 4. Material and Methods

The responses of the different EMD variants depend to a different extent on the local pattern details of realistic input images. To quantify the resulting deviations of the responses, we tested the models in simulation experiments.

### 4.1. Modelling

The different EMD models were implemented using C++. All components were realised as differential equations which were solved using the Euler method. The solver step size was 1 ms, *i.e.*, a sampling rate of 1 kHz was applied.

**Figure 3.**
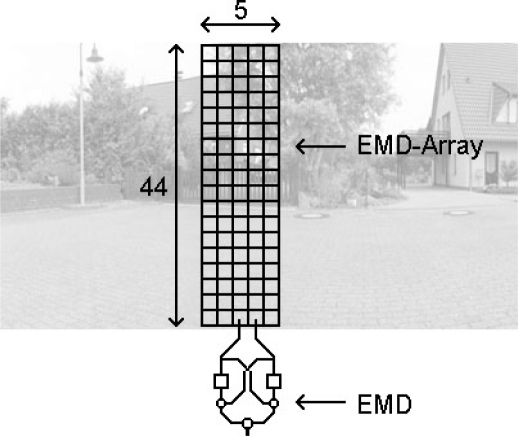
Moving EMD array. For the simulation tests an array of 44 × 5 input elements was used. Each element covers a 2° visual angle and applies a Gaussian shaped spatial low-pass filter (*σ* = 1.5°) to the input image. The simulated EMD array has a horizontal preferred direction, and consists of 176 EMDs. For the analysis, the EMD responses are spatially averaged. The observed intensity values of the input array change in time, depending on the horizontal velocity of the presented image.

For all tests, an array of 44 × 5 input elements was used. Each element covers 2° visual angle, thus the array covers a region of 88° (vertical) × 10° (horizontal). For each input element we applied a Gaussian shaped spatial low-pass filter at subpixel positions to the high resolution panoramic input image. The standard deviation of the filter mask was *σ* = 1.5°. This smooth subsampling method allows a continuous movement of the EMD array across the panoramic input image. Since each EMD operated on two horizontally neighbouring inputs, an array of 44 × 4 EMDs with a horizontal preferred direction resulted (Figure 3). The responses of the EMDs were spatially averaged. During the tests the array was shifted across the input images. Using 360° panoramic images allowed the simulation of a continuous motion in the input image for an extended period of time. The test images possess a high spatial resolution (10 pixels per degree visual angle), thus a high temporal resolution could be simulated.

The simulations were performed using different velocity regimes. For the constant velocity tests, eleven different velocities between 20°*/s* and 1,096°*/s* were used (20°*/s*, 29°*/s*, 44°*/s*, 66°*/s*, 99°*/s*, 148°*/s*, 221°*/s*, 330°*/s*, 492°*/s*, 735°*/s*, 1,096°*/s*). We also tested a stimulation with a sinusoidal speed profile.

The simulations were carried out for three different EMD versions: (1) h-l-EMD (Figure 1(a)), Equation (2) h-l-cc-EMD adding dynamic contrast normalisation (Figure 2(a)), and finally (3) h-l-scc-EMD with a simplified normalisation stage (Figure 2(b)). Since the results obtained for (2) and (3) are very similar to each other we mostly show results for variants (1) and (3) in the plots. All input images had a value range between 0 and 255.

### 4.2. Test Settings

#### Constant Velocity Tests

In the first tests, we examined the response of the models to a *perfect* sine pattern.

In addition we generated a panoramic sine pattern by video capture of a printed sine grating. This signal contains noise and local deviations from the perfect pattern caused by slight changes in illumination and saturation effects caused by the printer or camera. Thus the resulting pattern is no longer a perfect single wavelength pattern but has a broader frequency spectrum with a strong fundamental frequency. The spatial wavelength (16.36°) and intensity range (0–255) of the perfect sine pattern was matched to this panorama.

**Figure 4.**
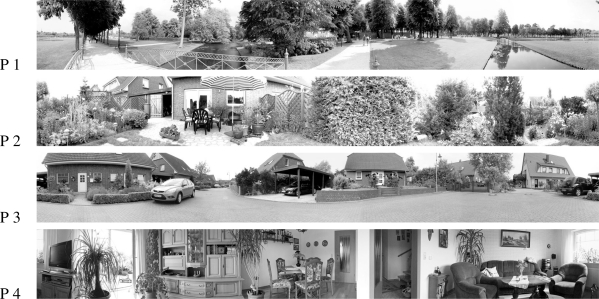
For the different tests four different panoramic images were used. These images where generated using photo stitching. The images have a size of 3,600 × 442 pixels, corresponding to 10 pixel/° horizontal resolution.

Additional tests examined the EMD array response to natural scenes (Figure 4) that were chosen to cover a variety of different environments. Four different panoramic images were used which were generated by stitching multiple photos, resulting in 3,600 × 442 pixel images. For the simulations, only the green channel was used. The histogram of brightness values of the resulting monochrome image was scaled to cover the full range of values (0–255), leading to a global Michelson contrast [31] of 100% (see Appendix B).

We compared the four panoramic images with regard to changes in the detector response. We looked for time-dependent changes in response amplitude, preferred pattern velocity, and time-dependent deviations from the average response. We plot the EMD array responses versus phase of the input pattern instead of versus time. This allows to compare the variations of the EMD array responses based on the phase of the input pattern for the different velocities. Additionally the influence of the average image contrast on the EMD array responses was examined. For this purpose we reduced the global contrast to values of 75%, 50% and 25% respectively.

#### Sinusoidal Velocity Stimulation

EMD responses are known to depend on acceleration and higher order temporal derivatives of the pattern velocity [13]. Therefore, the final test examines the response of the EMD arrays to stimuli moving with continuously changing velocity. The velocity varied sinusoidally. The peak velocity was switched between 400°/s and 100°/s. We tested different temporal frequencies for the sinusoidal velocity modulation. Here we present exemplarily data for 0.2 Hz and 4 Hz respectively, because these clearly illustrate the acceleration dependency of the EMD response. We used all four panoramic images and for each image we started at five different positions of the pattern and then calculated the average response.

### 4.3. EMD Parametrisation

The parameters of the different models were determined by systematic variation. The time constants of the high-pass and low-pass filters were set such that the resulting velocity curve showed a maximum at 100°/*s* for the panoramic image 1 (park scene, see Figure 4). Responses of fly motion sensitive neurons to similar stimulation typically show a peak approximately at this velocity [21,22].

The intention of this parametrisation was to obtain a similar velocity optimum of the different EMD arrays for the panoramic images. However, for the sine patterns this parametrisation results in different preferred velocities for the different models.

Only the high-pass filter constants at the input stage and the constants used for the delay elements (first order low-pass filters) were adjusted. The time constant of the low-pass filter used for the normalisation in the (s)cc models was set to fixed values (see Appendix A).

### 4.4. Analysis

For the analysis of the different models, the time-course of the responses of the EMD array was examined. The transient oscillation observed at stimulus onset [10] was excluded from the analysis. The duration of the examined EMD array response was chosen so that for all velocities the responses to the same pattern segment were examined, which makes it easier to differentiate between the consequences of local and global pattern modifications. For each velocity, the entire response was averaged and the standard deviation was calculated. The mean values were plotted versus velocities (velocity tuning curve).

### 4.5. Quality Criterion

To quantify the robustness of the response of the different models with respect to local pattern properties, the discriminability with respect to the velocities was quantified using Fisher’s linear discriminant value. In the following *X̄* is the averaged value of a measured signal *X* = {*x_t_*}, *t* = 1..*N_X_* where *N_X_* is the number of data points in the signal. The variance of the signal is defined as

(10) $$s_{X}^{2}=\frac{1}{N_{X}}\sum_{t=1}^{N_{X}}{\left(x_{t}-\bar{X}\right)}^{2}$$

Fisher’s linear discriminant criterion for two such signals *X* and *Y* is [32]

(11) $$J_{X,Y}=\frac{{\left(\bar{X}-\bar{Y}\right)}^{2}}{s_{X}^{2}+s_{Y}^{2}}$$

The criterion value increases with increasing distance between *X̄* and *Ȳ* and with decreasing variances. If both signals consist of nearly constant values, the criterion value approaches infinity.

In the tests, the EMD array responses to *n* different velocities *v_i_*, *i* = 1..*n* were measured. For each pair of neighbouring velocities *v_i_* and *v*_*i*+1_ we determined Fisher’s criterion *J*_*v*_*i*_,*v*_*i*+1__. A cumulative quality value is computed as average of the resulting *n* − 1 values

(12) $$Q=\frac{1}{n-1}\sum_{i=1}^{n-1}J_{v_{i},v_{i+1}}$$

We compared different EMD models with respect to their quality value. A higher quality value implies better discriminability of the responses to different velocities and fewer pattern noise effects.

## 5. Results

### 5.1. EMD Array Response to Sine Pattern

For the first test, the perfect sine pattern and the noisy panoramic sine image generated from video recordings of a printed pattern were used. The steady-state responses of the EMD arrays to the perfect sine pattern can be predicted by Equations (2) (see Section 2), (8) and (9) (see Section 3).

**Figure 5.**
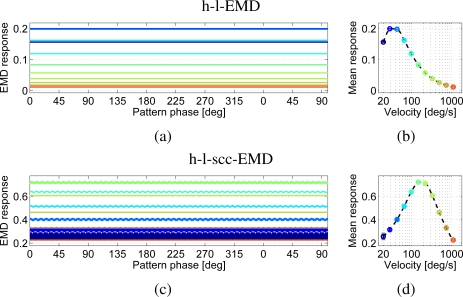
Responses of an h-l-EMD array **(a, b)** and an h-l-scc-EMD array **(c, d)** to a perfect sine pattern. (a) and (c) show the responses versus pattern phase for eleven different velocities after the initial response transient has faded away. (b) and (d) represent the velocity dependence of the mean response amplitude (logarithmic velocity axis). The dashed line represents the interpolated velocity curve. Markers indicate the mean values. Additionally the standard deviation is shown (too small to be visible here). (a, b) h-l-EMD array: The response shows the predicted nearly constant behaviour. (c,d) h-l-scc-EMD array: The response is oscillating. The amplitude of the oscillation is inversely correlated with the velocity.

The simulated responses of the h-l-EMD array show the predicted behaviour (Figure 5(a,b)), *i.e.*, a constant response over time. This behaviour is reflected in high quality values (Table 1). This means that small changes in pattern velocity can be observed almost directly as changes in the output signal. For the (s)cc-EMD array, oscillations around a constant mean value can be observed (e.g., h-l-scc-EMD in Figure 5(c,d). The amplitude of these oscillations is reduced with increasing velocity.

The sine pattern with noise has high and low frequency noise in pattern brightness. These imperfections introduce the above-mentioned additional Fourier components to the spectrum of the sine pattern. The resulting contrast is lower than in the perfect sine.

**Figure 6.**
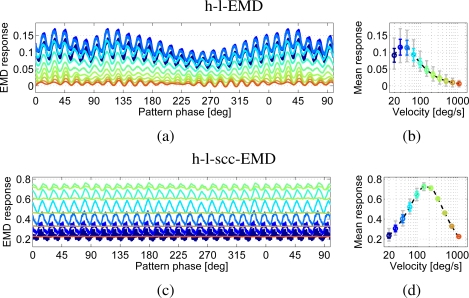
Responses of an h-l-EMD array **(a, b)** and an h-l-scc-EMD array **(c, d)** to a sine pattern with local noise and changes in local intensity. (a) and (c) show the responses versus pattern phase for eleven different velocities after the initial response transient has faded away. (b) and (d) represent the velocity dependence of the mean response amplitude (logarithmic velocity axis). The dashed line represents the approximated velocity curve. Markers indicate the calculated mean values. Additionally the standard deviation (coloured) and the minimum to maximum range of values (grey) are shown. (a, b) h-l-EMD array: The noise leads to a strong response modulation in comparison to the response to the perfect sine pattern. The different velocities cannot be distinguished as easily as is possible for the noise-free pattern. (c, d) h-l-scc-EMD array: The fluctuations in the response are reduced, especially the low-frequency ones, compared to those of the h-l-EMD.

**Figure 7.**
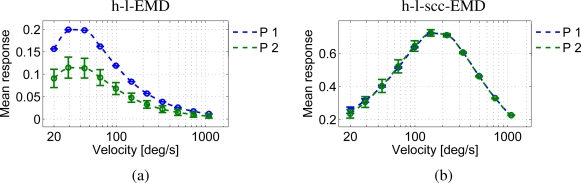
Velocity tuning of the different models for the perfect sine pattern (P 1) and the noisy panoramic sine pattern (P 2). **(a)** The h-l-EMD array shows strong differences in the responses whereas **(b)** the h-l-scc-EMD array shows only minor differences (curves are overlapping).

In comparison to the noise-free sine pattern, the h-l-EMD model shows strong time-dependent response modulations to the noisy camera generated sine pattern (Figure 6(a,b)). This results in higher standard deviations of the time-dependent signals from the mean response and a high minimum to maximum range of response values (grey line). The modulations in the EMD array response reflecting high and low frequency noise in pattern brightness lead to low quality values (see Table 1). In addition, the mean response level at a given velocity was consistently lower than the corresponding mean value of the response to the perfect sine pattern (Figure 7(a)). The mean response as averaged over a longer period allows us to distinguish between the different velocities (Figure 6(b)). However, local mean values based on averaging over a narrow time window would differ from the global mean value. Based on only a short averaging time this distinction would not be possible. Only direction detection is then possible.

The (s)cc-EMD array shows a more robust response behaviour with respect to pattern noise (Figure 6(c,d)). The response oscillates with a larger amplitude than that to the perfect sine pattern, but especially when compared to the responses of the h-l-EMD array, the low-frequency response fluctuations are largely eliminated (Figure 6(c,d)). The mean response levels show no significant changes when compared to the results with a perfect sine pattern (Figure 7(b)). Also, the local mean values are independent from image noise. Only the standard deviations are increased. The amplitude of the oscillations superimposed on the steady-state response decreases with increasing velocity (Figure 6(d)).

### 5.2. EMD Response to Different Natural Scenes

Four different natural scene images were tested (Figure 4). The EMD arrays were shifted across these images with eleven different constant velocities.

**Figure 8.**
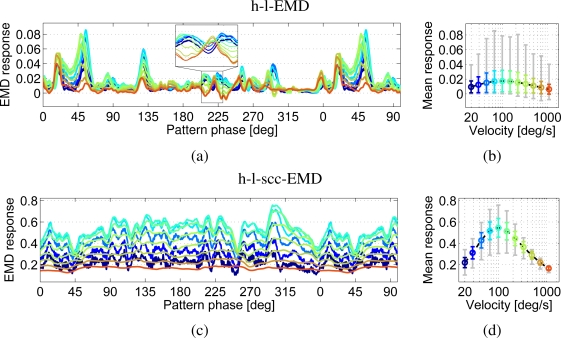
Time dependent responses to a natural panoramic image (see Figure 4 no. 1, park scene) moving at different constant velocities (**a** and **c**), and velocity dependence of averaged responses with standard deviation (coloured) and the minimum to maximum response range (grey) (**b** and **d**). (a, b) h-l-EMD array and (c, d) h-l-scc-EMD array. The h-l-EMD array shows a strong pattern dependency. The responses of the (s)cc-EMDs arrays show a smaller pattern dependency which is reduced for larger velocities.

**Figure 9.**
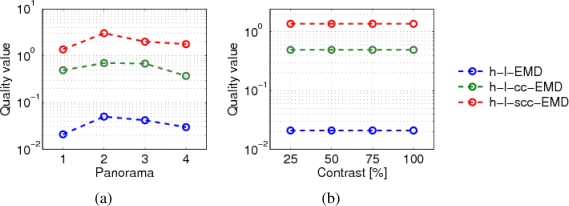
(a) Quality values for the response to natural panoramic images (see Figure 4, nos. 1–4). (b) Quality values of the different variants of EMD arrays for the responses to varying contrast of the panoramic image 1 (see Figure 4, no. 1, park scene). A large quality value indicates a better discriminability of responses to different velocities. Note the logarithmic scales of the quality axes of the diagrams.

The response of the h-l-EMD model strongly depends on local pattern properties (Figure 8(a)). This is reflected in the pronounced response modulations (Figure 8(b)). For example, at 50° the h-l-EMD shows significant response modulations which are similar for all tested velocities (Figure 8(a)). Additionally the response modulations show an asymmetry of the value range (Figure 8(b) grey line). While the responses show large deviations for values above the mean response values, the values below the mean response values lie only in a small range. The large response modulations result in a small quality value (Figure 9(a)). Again, averaging over the entire response obtained for each of the different velocities reveals a distinct velocity dependence. However, if the averaging time window is too small, the different velocities can not be distinguished. The responses are not consistently separated from each other. Responses to velocities associated with a large average response are small in certain pattern positions (Figure 8(a) inset). Thus, only direction detection is possible, but with a strong pattern noise influence which can even lead to false direction detection (e.g., negative values at 75°).

The response behaviour of the (s)cc-EMD arrays is more robust against changes in the pattern properties (Figure 8(c,d)). The standard deviations of response fluctuations around the mean are smaller than those of the h-l-EMD model, and the responses are characterised by a more symmetrical value range. This is also reflected in the corresponding quality values (Figure 9(a)), which are significantly larger for the (s)cc-EMDs than for the h-l-EMD model, emphasising the increased insensitivity to local pattern properties. Furthermore, except for velocities close to the peak of the velocity curve, the responses to the different velocities show a constant magnitude relation (Figure 8(d)). The quality values for the h-l-scc-EMD are even higher than the ones for the h-l-cc-EMD. Thus the averaging window necessary for velocity differentiation is supposed to be smaller for the h-l-s(cc)EMDs than for h-l-EMDs.

**Figure 10.**
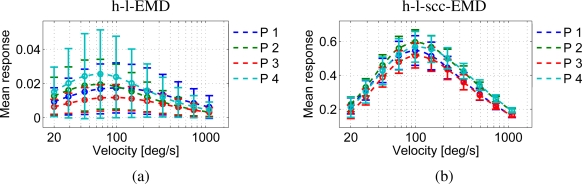
Velocity tuning curves of the h-l-EMD array and the h-l-scc-EMD array for all four panoramic images (P1–P4, see Figure 4). Changing the pattern strongly affects the amplitude of the response peak in the h-l-EMD model (**a**). For the h-l-scc-EMD array (**b**), the differences between responses to different panoramic images are less pronounced, but a small variability of the response amplitude remains.

The responses of the h-l-EMD model to the four panoramic patterns differ in amplitude and standard deviation. Figure 10(a) shows the velocity tuning curves of the h-l-EMD model. Also the (s)cc-EMD is pattern-dependent, but the velocity tuning curves are more similar to each other with respect to the maximal amplitude, the position of the response peak, and the standard deviation (Figure 10(b)). For all models and panorama images, the quality values are similar (Figure 9(a)).

**Figure 11.**
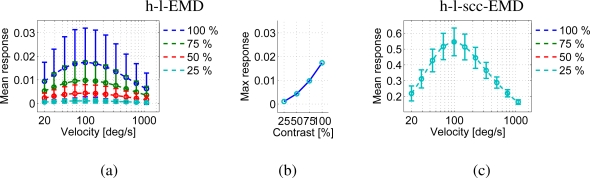
Velocity tuning curves of the h-l-EMD array and the h-l-scc-EMD array for different global contrasts. The contrast of the park scene panorama (Figure 4, no. 1) was set to 100%, 75%, 50%, and 25% respectively. The response of the h-l-EMD array (**a**) shows the quadratic contrast dependence (**b**). In the responses of the h-l-scc-EMD (**c**) the contrast dependency is eliminated almost completely (curves are identical for all contrast values).

We varied the global contrast of the panoramic images. The h-l-EMD model shows the predicted quadratic contrast dependence (Section 2, Figure 11(a,b)). The predicted contrast independence of the (s)cc-EMD model is also verified by our simulation results (Figure 11(c)). The amplitudes of responses of the (s)cc-EMD array are contrast independent for all pattern velocities. The quality values of all models are contrast independent (Figure 9(b)). The quality values of the (s)cc-EMDs are higher than of the h-l-EMD model.

### 5.3. Dynamic Change of Velocity

It has previously been shown that the EMD responses do not only depend on velocity, but also on higher order temporal derivatives of velocity, most importantly, on acceleration [13]. We therefore performed dynamic tests, in which the EMD array was shifted with a sinusoidally modulated velocity across the input images. For the tests, the velocity was varied sinusoidally with maximum velocities *v_max_* = 100°/s, and *v_max_* = 400°/s. The frequency of the velocity modulation was either *f_v_* = 0.2 Hz, or *f_v_* = 4 Hz, to assess the effect of the resulting accelerations.

**Figure 12.**
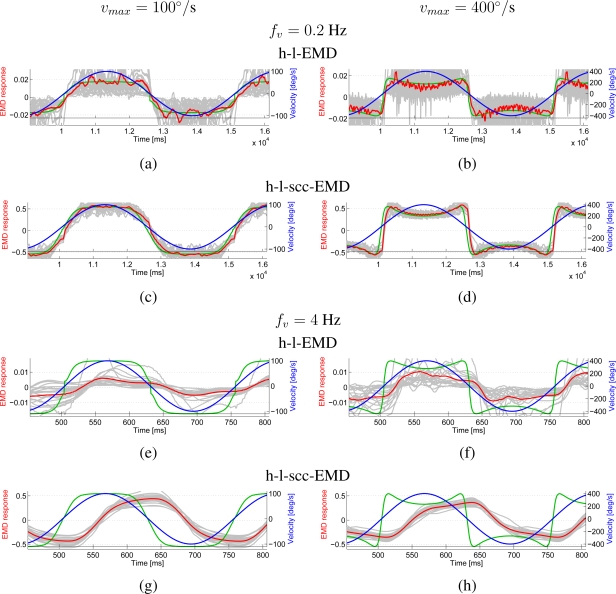
Responses of the EMD arrays to dynamic stimulation. Blue curves and right scales show the stimulus velocity versus time, red and grey curves and left scales show the responses. Grey lines represent individual simulation results, red lines average results for 4 panorama images and 5 different starting positions. The green curves show the theoretical response predicted from the steady state response curves derived from the previous tests. The velocity of panorama images was oscillated sinusoidally at 0.2 Hz (a–d) or 4 Hz (e–h). The peak velocity was 100°/s (left column) or 400°/s (right column). Responses of two model variants are shown: h-l-EMD (a,b,e,f) and h-l-scc-EMD (c,d,g,h).

We used all four panoramic images as input patterns and started the simulated movement at five different locations in the image, resulting in 20 different input signals altogether. Based on the individual responses (Figure 12, grey lines) we calculated the average response (red line).

We also plot a theoretical response predicted from the steady state response curves derived from the previous tests (green line). With *v_max_* = 100°/s the predicted response reflects the velocity monotonically, but the nonlinear tuning of the detector results in a compressive deformation of the response when compared to the sinusoidal time course of velocity. In case of *v_max_* = 400°/s, the response predicted from the steady-state tuning shows a fall-off of the response for velocities exceeding the optimal velocity of 100°/s.

For the first tests with *f_v_* = 0.2 Hz, the averaged responses (red line) of the h-l-EMD array can, in a first approximation, be derived from the steady-state tuning (Figure 12(a,b) green lines matching the red ones). The individual responses (grey) show a strong pattern dependency and, for some of the panoramic images, the response hardly reflects the movement.

For the h-l-scc-EMD in both tests (Figure 12(c,d)) the averaged response (red line) can also be predicted from the steady-state tuning (green line), including the response fall-off at higher velocities. Compared to the situation in the h-l-EMD, the individual responses of the h-l-scc-EMD are also more similar to the predicted response and show only a minor pattern dependence.

For the tests with a frequency of 4 Hz, a similarity to the predicted response can not be observed either for the individual responses of the h-l-EMD array nor for the average response (Figure 12(e,f)). The responses show a consistently lower amplitude compared to the predicted signal. The predicted fall-off at higher velocities is not reflected in the observed response.

The robustness of the responses of the h-l-scc-EMD is less affected by the higher velocity dynamics (Figure 12(g,h)). As in the low-dynamic tests, the individual responses of the h-l-scc-EMD show smaller deviations from the average response than those of the h-l-EMD. The response amplitude is less prominently reduced compared to the predicted response. However, the predicted fall-off of the response at higher velocities is only weakly observable.

The high-dynamic tests clearly show that dynamic responses of EMDs cannot be explained adequately by steady state velocity tuning. Rather, the responses of arrays of EMDs depend on a combination of pattern velocity and its higher order temporal derivatives [13]. Since this is a general property of the detection mechanism and unrelated to pattern contrast, the (s)cc-EMDs do not show a qualitative improvement in this respect.

For both models, the change of sign in the observed response is delayed with respect to the change of sign in the prediction based on the velocity tuning. For the low-dynamic tests (*f_v_* = 0.2 Hz) this delay seems to be shorter than in the high-dynamic situation (*f_v_* = 4 Hz). Note, however, the different scaling of the time axis of the plots.

The delay of the h-l-scc-EMD array is significantly larger than that of the h-l-EMD array. This can be attributed to the phase shift caused by the additional low-pass filters L*_w_* in the contrast-normalising circuit of the h-l-scc-EMD.

## 6. Discussion

Flying insects use optic flow information for course stabilisation, obstacle avoidance and navigation. They extract and analyse this information in their tiny brain using a relatively computationally cheap process [6,7,9]. This process is based on local motion estimates computed in elementary motion detectors (EMDs). In a subsequent step, these local motion estimates are spatially integrated by large field neurons which are assumed to implement a set of matched filters for certain optic flow patterns. This filter-based architecture, though applied to local velocity, estimates computed by computer-vision algorithms, was successfully applied to mobile robots [14,15].

In contrast to these algorithms, the insect-inspired EMD encodes the image velocity in a nonlinear and ambiguous way which complicates the technical application of the EMD principle. The EMD response peaks at a certain velocity and decreases for velocities below as well as above this optimum [8]. Furthermore, the EMD shows a strong modulation in its response which is caused by three factors:

- the response of the detector depends on the spatial wavelength of the input image, which is a minor issue for natural images composed from a broad spatial spectrum, (ii) the response depends not only on the velocity but also on its higher temporal derivatives, most prominently the acceleration, (iii) mathematical analysis reveals a quadratic dependence of the basic EMD response on contrast. However, experimental results in flies show this quadratic dependency only for very low contrast values. For higher contrast values the response becomes contrast-independent due to saturation nonlinearities and adaptive elements in the visual pathway [10].

This discrepancy between the model and its biological counterpart can be reduced by adding saturation nonlinearities to the EMD circuit and extending the model with additional spatial and temporal filters which are either experimentally characterised in the insect motion pathway or are at least biologically plausible [24,25]. On the one hand, using these extensions, the EMD responses are much less sensitive to contrast changes in the stimulus pattern. On the other hand, these augmentations add computational overhead and additional free parameters to the algorithm.

In this study we do not aim at a plausible model for the insect visual system but seek to make the computationally cheap principle of correlation-based motion detection applicable for mobile robot control. We present an augmentation of the EMDs, the (simplified) correlation coefficient EMD (h-l-(s)cc-EMD) which reduces the response modulation caused by local changes in pattern contrast. This is achieved by a dynamic contrast normalisation of the response by means of linear filters and simple static nonlinearities (square, square root, division). With this contrast normalisation, we can eliminate pattern noise resulting from local changes in contrast and local average luminance.

We do not address the ambiguity of the EMD response, its dependency on the spatial spectrum of the stimulus, or the acceleration. Like the basic EMDs, the (s)cc-EMDs have a nonlinear and ambiguous velocity tuning with a preferred velocity causing the maximal response. Although problematic on the first glance, this property is not necessarily a drawback of the mechanism. The resulting signal compression can be advantageous in the context of sensor signals with a limited range. It was also observed that such a sensor response can increase controller stability [33].

Although the (s)cc-EMD is not meant to model the circuits in the fly brain, the normalisation mechanism is constructed from system theoretic elements that are also employed to account for the functional properties of neuronal circuits. In this sense, our EMD model with dynamic contrast normalisation is a biologically plausible model. Nevertheless the contrast independency implemented in the (s)cc-EMD is too strong to match observation in biological systems. Models employing a saturation nonlinearity for signal compression [10,24,25] are generally better suited to fit biological data.

We have shown in an analytical way that the responses of our new EMD models are largely independent of contrast. However, the response of the new model shows an oscillation depending on the temporal frequency of the input pattern.

In model simulations we compared the response behaviour of an array of basic EMDs with an array of our novel (s)cc-EMDs using sinusoidal stripe patterns as well as natural images. The simulations with a noisy sine pattern show that the pattern noise in the response of the basic model is stronger than in the contrast normalised EMDs. Although the (s)cc-EMDs show an oscillation behaviour, the dependence on local pattern properties is largely reduced. The amplitude of the additional oscillation is small compared to the amplitude of the modulation caused by local pattern structure.

Tests on panoramic photo stimuli of visually complex scenes show that the responses of the EMD array with dynamic contrast normalisation are significantly less dependent on pattern properties compared to the basic EMD. The (s)cc-EMD array shows a high robustness in the mean response behaviour independent of the specific panoramic scenes. As a consequence, these models signal changes in velocity more reliably in visual environments relevant for the technical application of these sensors. It could be shown that the robustness of the (s)cc-EMD array increases with increasing velocities. Consequently, the (s)cc-EMD may be especially suited for a system operating in the super-optimal part of the velocity tuning curve.

Furthermore, we examined the EMD array responses to continuously changing velocities. For this test we used a panoramic pattern which moved with sinusoidally modulated velocities. The additional filters slightly increase the temporal latency of the response to velocity changes. This drawback is outweighed by a considerable increase in robustness of the responses. Response components caused by accelerations are not amplified by the normalisation process as the tests with higher frequency sinusoidal velocity changes indicate. The deviations of responses to individual panorama images from a response averaged across different patterns is far less pronounced in the (s)cc-EMD responses compared to those of the h-l-EMD.

The elements used in the (s)cc-EMDs are easily transferable to digital or analog hardware solutions or real-time systems based on digital signal processors.

First preliminary results obtained with the (s)cc-EMD as an input to a simple saccadic obstacle avoidance mechanism proposed earlier [19] show that such a system is much less sensitive to changes in the textural properties of the environment compared to a system based on basic EMDs. Detailed analysis of obstacle avoidance based on (s)cc-EMD sensors will be presented in a forthcoming study.

## Figures and Tables

**Table 1.** Quality values *Q* for the different models (large value indicates a better discriminability of responses to different velocities). It can be seen that for the basic model the additional noise and the local intensity changes in the real sine pattern have a strong effect on the output. The (s)cc models show smaller changes in the quality of the response. The quality for the real sine pattern is much better in the (s)cc models.

	basic model	correlator models	
	h-l-EMD	h-l-cc-EMD	h-l-scc-EMD

perfect sine	5.4 × 10^25^	849.7	3 × 10^04^
realistic sine	0.715	12.88	494.8

## A. Parametrisation

As mentioned in Section 4.3, the models are parametrised by systematic parameter variation. The criterion for parameter selection was that the velocity curve on the panoramic images shows a maximum at 100°*/s*. This resulted in the time constants *τ*_*L_d_*_ and *τ*_*H_p_*_ shown in Table 2.

The time constant of the normalisation part of the (s)cc models *τ*_*L_w_*_ does not influence the position of the peak in the velocity tuning. For application, it is important to note that a large time constant *τ*_*L_w_*_ results in a slower reaction to changes in the velocity while small values *τ*_*L_w_*_ reduce the contrast independence (data not shown). Due to this tradeoff, the choice of *τ*_*L_w_*_ depends on constraints of the application.

**Table 2.** The time constants used for the different filters in the examined EMD models.

model	parameter	value [ms]

h-l-EMD	*τ_H_p__*	140
	*τ_L_d__*	120

h-l-cc-EMD	*τ_H_p__*	25
	*τ_L_d__*	20
	*τ_L_w__*	36

h-l-scc-EMD	*τ_H_p__*	15
	*τ_L_d__*	15
	*τ_L_w__*	36

## B. Contrast Variation

The contrast *C* of the monochrome input image is measured using the Michelson formula [31]:

(13) $$C=\frac{L_{\max}-L_{\min}}{L_{\max}+L_{\min}},$$

Where *L_max_* and *L_min_* are the maximum and the minimum grey value of the image. For the images scaled to the full range of values (0-255), we defined the contrast as *C*_100_ = 100%. To reduce the contrast, *L_max_* and *L_min_* are adapted by shifting

(14) $$C_{x}=\frac{x}{100}C_{100}=\frac{\left(L_{\max}-d\right)-\left(L_{\min}+d\right)}{\left(L_{\max}-d\right)+\left(L_{\min}+d\right)}.$$

The pixels are scaled to the range between *L_max_* − *d* and *L_min_* + *d*.
